# Memory Detection 2.0: The First Web-Based Memory Detection Test

**DOI:** 10.1371/journal.pone.0118715

**Published:** 2015-04-13

**Authors:** Bennett Kleinberg, Bruno Verschuere

**Affiliations:** 1 Department of Clinical Psychology, University of Amsterdam, Amsterdam, The Netherlands; 2 Department of Psychology, Ghent University, Ghent, Belgium; 3 Department of Clinical Psychological Science, Maastricht University, Maastricht, The Netherlands; Centre de Neuroscience Cognitive, FRANCE

## Abstract

There is accumulating evidence that reaction times (RTs) can be used to detect recognition of critical (e.g., crime) information. A limitation of this research base is its reliance upon small samples (average *n* = 24), and indications of publication bias. To advance RT-based memory detection, we report upon the development of the first web-based memory detection test. Participants in this research (Study1: *n* = 255; Study2: *n* = 262) tried to hide 2 high salient (birthday, country of origin) and 2 low salient (favourite colour, favourite animal) autobiographical details. RTs allowed to detect concealed autobiographical information, and this, as predicted, more successfully so than error rates, and for high salient than for low salient items. While much remains to be learned, memory detection 2.0 seems to offer an interesting new platform to efficiently and validly conduct RT-based memory detection research.

## Introduction

Lie detection can count on great attention from the public, as well as from researchers. Polygraph tests and voice stress analyses tests are widely applied, but remain highly controversial. In recent years, deception researchers have turned gears and switched from a focus on stress to a focus on cognition [1]. Cognition-based lie detection searched for cognitive differences between lying and truth telling. With the focus on cognition, there is also a renewed interest in reaction time (RT) measures [2]. Reaction times provide a means to tap into the cognitive complexity of stimulus processing. As such, reaction times have been used to index the recognition of critical (e.g., crime) information and serve as memory detection test.

### 2.1. Memory detection

Memory detection testing has also been referred to as Guilty Knowledge Testing [3] or Concealed Information Testing [4]. This nomenclature encompassed a family of techniques that share the same central goal and methodology. The goal is to assess through indirect measures whether the examinee recognises certain information. The methodology involves the presentation of the critical piece of information within a series of very similar, yet non-critical pieces of information. For example, in a murder investigation, memory detection could involve the presentation of the murder weapon (a knife) among a series of possible murder weapons (a gun, a hammer, a trophy, an axe). When using properly matched alternatives, memory detection provides good protection for the naive examinee, because he/she cannot recognise the critical item from the other items. The recognition of the critical item by the knowledgeable examinee, on the other hand, will result in a differential response as compared to the non-critical items. Memory detection has been widely researched in the laboratory [5][6], and is regularly used in criminal investigations in Japan [7]. Originally, memory detection was exclusively based upon skin conductance measurement. Later on, researchers have used other physiological measures, as well as electrophysiological, neural and behavioural measures, including reaction times (RTs).

### 2.2. Memory detection using reaction times

Reaction times are an attractive measure from an applied point of view. They do not require the technology and sophistication of physiological measures. Memory detection with reaction times requires a single computer and can be completed in less than 15 minutes. Such a test involves the presentation of the critical item, called *probe*, within a series of non-critical items, called *irrelevant items*, on the computer screen. Participants are required to press a button as fast as possible upon encountering the stimuli, and a response deadline is often used to assure immediate responding. Simple button pressing, however, may not to lead to robust reaction time differences [4]. One needs to assure that the examinee has sufficiently processed the stimulus [8]. The typical behavioural instruction is to press one button for a dedicated set of items, called *targets*, and to press another button for all other stimuli. Building on the sample above, the murder suspect may be asked to press YES for the target item ‘stick’, and NO for all other items (‘knife’, ‘gun’, ‘hammer’, ‘trophy’, ‘axe’). Slower responding to the probe item compared to the irrelevant items is taken as an indication of crime recognition. Seymour, Seifert, Shafto and Mosmann [9] were the first to show that RT-based memory detection can be highly successful; a finding this group has replicated several times (see e.g., [10], [11], [12]). Research from labs in Romania [13][14][15], North America [16], Belgium, and The Netherlands [17][18] confirms the potential of reaction times as a stand-alone memory detection measure. A limitation of this research base, however, is its reliance upon small samples, and the indications of publication bias [19].

### 2.3. Power

A recent meta-analysis on the validity of reaction times to detect deception [19] included 24 memory detection studies that reported results of reaction times of 583 participants. This implies an average sample size of *n* = 24. The recent crisis in psychological research [20][21][22] has raised renewed attention to power and replicability. Statistical power depends on the significance level of statistical testing, the sample size and the effect size. A matter of concern is that most effects in psychology are of moderate effect size, while most psychology studies do not have sufficiently large sample sizes to detect such effects [23]. Given the large effect sizes in the field of memory detection in general [5][6] and RT-based memory detection in particular (*d*_*within*_ = 0.895 [95% CI: 0.759, 1.030] after correction for publication bias, see [19]), it could be argued that memory detection studies with 24 participants are sufficiently powered to observed the probe-irrelevant difference. An effect of *d*_*within*_ = 0.895 can be detected with less than 20 participants with high power. However, memory detection is typically no longer interested in the demonstration of the basic phenomenon (RT_probe_> RT_irrelevant_), but in possible moderators such as the saliency of the test items, leakage of critical information to naive participants, attempts to fake the test, and individual differences in detectability [2]. For these moderators, effect sizes are more likely to be of moderate size [23]. For decades, methodologists and statisticians have recommended the use of larger sample sizes [24][25]. Still, sample sizes in psychological research have not systematically increased in the last 50 years [26]. One reasons for this stagnation is that researchers face practical constraints and their limited resources (money, lab space and availability, research assistants) do not always allow to conduct research in large samples. In this paper we introduce memory detection 2.0, a web-based memory detection solution that will allow conducting memory detection research with large samples. At the same time, online memory detection is quicker and less expensive than laboratory research. Moreover, online research is more transparent. As will become clear, by providing the link of our online memory detection studies, we maximise reproducibility, as everyone with a computer connected to the web will be able to visualise the method used in our studies.

### 2.4. Online Testing

Our idea is to make use of the potential offered by online testing. Behavioural researchers are increasingly using the web for surveys, and more and more also for other behavioural studies [27]. Crump, McDonnel and Gureckis [28] examined the possibility to run RT experiments on the web using Amazon’s Mechanical Turk. They replicated several well-known cognitive phenomena, such as the Stroop effect whereby greater RTs are found for incongruent trials (i.e., the word “green” in red colour) than for congruent (i.e., the word “green” in green colour), and concluded ‘Even for extended experiments requiring problem solving and learning, and precise millisecond control for response collection and stimulus presentation, the data seem mostly in line with a laboratory results so long as the experiment methods were solid’. Still, Crump et al. [28] also discussed disparities between web-based and laboratory research, and noted that timing may vary with the participants’ web-browser. Apart from technical challenges, another disadvantage of online testing may arise from the fact that one has less control on whether the examinee understands the task, takes the task seriously and pays sufficient attention to the task, which may imply loss of quality in the data (but see [29]; [30]; [28]; [31]; [32]; [33]). While online testing has great potential in terms of efficiency, there is reason for caution and the validity of online RT-based memory detection needs to be determined empirically.

### 2.5. The present study

We had two main aims with this study. First, we wanted to develop a flexible web-based memory detection test that can be run in any web browser without installing plug-ins or buying licensed software. We investigated whether we could replicate the basic memory detection phenomenon observed in offline research (memory detection 1.0), that is, a large probe-irrelevant difference in RTs in knowledgeable, but not naïve participants. Second, we wanted to use this new platform to conduct well-powered studies on possible moderators in memory detection. In the present study we focused upon item saliency. Mock crime studies found that central details of the crime are better remembered and better detected than peripheral crime details [34][35][36][37]. Detection efficiency for high salient autobiographical details is also higher than that for low salient autobiographical details [38]. Thus, we expected RT-based memory detection to be more successful for high salient than for low salient details.

## Experiment 1

The study was approved by the ethical committee of the Department of Psychology of the University of Amsterdam (2013-CP-3053).

### 3.1. Pilot Studies

We conducted four pilot studies (sample sizes ranging from 25 to 55). The purpose of these pilot studies was to assure functionality on different browsers and operating systems, debug, and explore the possibility to perform online memory detection research. Apart from technicalities, these pilot studies taught us a few things. First, participants may be reluctant to reveal their true identities on the web for privacy reasons. This implied that we could not use certain items that are often used in memory detection research (first name, last name, names of close relatives, phone number, social security number, address). Second, the web provides great diversity in cultural and ethnic backgrounds, requiring additional consideration (e.g., how to match first names; whether favourite alcoholic drink is an appropriate item). Third, to prevent spelling errors (which can typically be checked and corrected in lab-based research), we turned to a predetermined list of items using drop-down menus. Fourth, in the current set-up of our online memory detection test, there is no online support (e.g., helpdesk). We therefore tried to adjust the procedure to maximise the chance that participants understand and follow instructions. Importantly, we implemented three successive practice phases that build up in complexity allowing the participants to become familiar with the requirements and speed of the task. Also, these practice phases were repeated until certain criteria were met (detailed below).

### 3.2. Materials and Method

#### 3.2.1. Participants

We opened up 250 HITs (see Procedure), but due to simultaneous starting times, 255 participants completed the test. There was no data for 3 participants, most likely due to the usage of very old versions of browser or operation system. Thus, we had data of 252 participants (56% females; *M*_*age*_ = 33.80 years, *SD*_*age*_ = 10.90). Participants received 0.5 $ as compensation. Fifty-two per cent of the participants indicated that they have obtained at least college education, 5% professional training, 12% of the participants completed high school, and 28% of the participants completed university education. The most common native language was English (61%), followed by Indian languages (27%), other languages (12%) and French and Spanish (each 0.4%). Participants originated mostly from the US (57%) and India (36%), but also from 8 other countries (Albania, Canada, France, Ghana, Greece, Italy, Myanmar, and Russia, together 7.4%). We recorded IP addresses and took a very conservative approach to exclude data of all 6 double IP addresses (12 exclusions, leaving *n* = 240). As further exclusion criterion, we excluded participants whose response error rate was high, that is when they had 50% or more errors on any of the three stimuli types (probes, irrelevants, targets). This criterion ensured that only those participants who understood the instructions were included in the final sample. Thirty-seven participants were excluded based on the error criterion, leaving *n* = 203. Total subject loss was 20.39%. The final sample consisted of 203 participants of whom 88 participants had been randomly assigned to the knowledgeable condition (57% females, *M*_*age*_ = 36.53 years, *SD*_*age*_ = 12.50) and 115 participants to the naive condition (51% females, *M*_*age*_ = 33.49 years, *SD*_*age*_ = 9.75). The sub-samples in the two conditions did not differ in gender *X* ^*2*^(1) = 0.41, *p* = .523, or age *t*(201) = 1.93, *p* = .052.

#### 3.2.2. Procedure

The experimental task was programmed and designed for web-browser application in Javascript/Jquery. The original experimental tasks can be found at http://www.lieresearch.com/?page_id=603 (Exp. 1) and http://www.lieresearch.com/?page_id=613 (Exp. 2).

The study was administered via Amazon Mechanical Turk (MTurk; see https://www.mturk.com/mturk/welcome), a website that allows individuals or businesses to post tasks (called HITs). These HITs can be completed by individuals registered to MTurk (MTurkers), and based upon their performance, the HITs are either approved or rejected (e.g., when not meeting certain quality checks). We advertised our study as a ‘15 minute lie detection study’, allowing participation of MTurkers who completed at least 95% of their previous HITs. The average duration of the study was 12 minutes. All participants agreed to an informed consent before they could participate. After providing demographic information the participants had to indicate autobiographical details (the probes). Participants indicated their birthday using a drop-down list containing all possible birthdays (e.g., 28 June), their country of origin using a drop-down list of all 252 countries (e.g., Argentina), their favourite colour using a drop-down list that contained 17 single word colours (e.g., Yellow) and their favourite animal out of 31 animals (e.g., Elephant). The colour list was derived from the most popular options reported in a survey that asked respondents for their favourite colour (http://awp.diaart.org/km/tur/survey.html) and supplemented by options we used in previous studies [39]. For the animals we chose popular one-word animals from a survey that asked respondents for their favourite animal (http://www.favoriteanimal.com/?fulllist=1). We tried to spread the available options across species. For instance, we used the word DOG for all dog races. Furthermore, participants were asked to indicate one another significant birthday, country, colour, and animal using the same answer options. This information was used to optimise stimulus selection (i.e., options that were listed as also being of personal significance were deleted from the list of predetermined irrelevant and target items and replaced).

After providing autobiographical details, participants were introduced with the memory detection task. They were told that they had to hide their true identity and pretend to be someone else (cf [18]). The details of the “fake identity” (the targets) were provided (e.g., 3 March, Norway, Purple, Tiger) and participants were instructed to learn these details. At their own time, participants continued to the target check that required to type in their new identity. In case of errors, participants were sent back to the target memorisation phase until they typed in all four details correctly. Once they successfully recalled the targets, participants began with the first of three practice phases of the memory detection test until the error criterion was met. After they completed the third practice phase they proceeded to the full test. After the full memory detection test, participants were asked to rate 12 autobiographical categories (see Table 1) on personal significance. Finally, participants were thanked and had the opportunity to leave a comment.

**Table 1:** Relevance Ratings of Different Categories of Autobiographical Information in Study1 (presented in descending order).

Category	*Mean*	*SD*
First name	7.16	2.57
Country of Origin	7.08	2.44
Birthday (Day and Month)	6.99	2.34
Favorite hobby	5.94	2.39
Favorite music	5.87	2.44
Favorite color	5.61	2.24
Favorite animal	5.48	2.50
Favorite dish (main course)	5.32	2.40
Favorite city (world wide)	5.17	2.49
Favorite movie	4.85	2.38
Favorite ice cream	4.35	2.49
Favorite soft drink	4.27	2.46

The between-subject experimental manipulation was that participants were randomly assigned to either the naive or the knowledgeable condition. In the naive condition they were not presented with their true autobiographical details (the probes) in the memory detection test. A set of 4 predetermined irrelevant items (PINK, 16 OCTOBER, BULGARIA, HORSE) served as probes. In the knowledgeable condition, the probes were the participant’s true autobiographical details.

Participants had to respond to the question “*Do you recognise this stimulus*?” by pressing either the E button for YES, or the I button for NO on their keyboard. The question and the response keys remained on the screen during the whole test as a reminder. The instructions stated that they had to respond with YES only to their new “fake identity” and NO to all other stimuli. Each trial in the task consisted of one autobiographical detail (e.g., IRELAND) being displayed as a word in the middle of the screen for exactly 1500ms. If the participant’s response was incorrect, that is she responded with NO to target items or with YES to probe or irrelevant items, the word WRONG appeared below the stimulus in red colour for 200ms. If the response did not happen within the response deadline of 800ms, the message TOO SLOW appeared in red colour above the stimulus for 200ms. We recorded reaction times from the onset of the stimulus on the screen until one of the two response keys was pressed. RTs were recorded using the *performance*.*now* method in JavaScript, which provides timing accuracy in microseconds, and contrary to the *Date*.*now* method operates independently of the users’ system clock. This might solve some of the possible technical problems mentioned by Crump et al. [28]. At the bottom of the screen was a progress bar that showed the user’s progress during the experimental task. After a response key was pressed or the 1500ms presentation time elapsed, the next stimulus appeared on the screen, resulting in a maximum response time of 1500ms. The stimulus appeared in a 10 millisecond-long fade-in animation and faded out in variable inter-stimulus interval (ISI). The ISI between two trials was either 250ms, 500ms, or 750ms.

All word stimuli were presented in the CIT-usual 1:1:4 ratio, that is of the total 240 trials in the full test, 40 were probe stimuli, 40 were target stimuli, and 160 were irrelevant stimuli, so that each stimulus was displayed exactly ten times (or one tenth of it for the practice phases, respectively). The sequence of stimuli was semi-randomised in a way that there were 10 blocks that each consisted of the 24 unique stimuli which prevented consecutive repetition of stimuli within one block. The same procedure was used for randomisation with the three ISIs. In order to ensure that the task was understood properly and instructions were clear, each participant had to pass through a stepwise practice procedure that allowed the participants to become used to the speed and requirements of the task. Each of the three practice phases consisted of 24 trials. In the first practice phase participants could pace the speed of the trial sequence themselves, so that the stimulus disappeared only after a response key was pressed. In this phase they received WRONG feedback, but not TOO SLOW feedback. In the second practice phase, the 1500ms stimulus display time was added. There was WRONG feedback, but still no TOO SLOW feedback. The last practice phase was identical to the full test with TOO SLOW after the response deadline and WRONG feedback. Before each practice phase they were instructed on how to respond and told that the speed of the test will increase with each practice phase. After each practice phase they received a feedback based on their performance (e.g., “Try to be faster and remember the instructions”) and could only proceed if their mean reaction time was faster than 800ms and if their target accuracy was at least 50%. If they failed to meet these requirements they had to do the respective practice phase again until their performance was satisfactory. We built in the target error criterion in the practice phases to ensure proper understanding of the instructions.

We used birthday and country of origin as high-salient and favourite colour and animal as low-salient autobiographical details. The choice of the categories birthday (*M* = 6.55, *SD* = 1.94), favourite colour (*M* = 4.72, *SD* = 2.36) and favourite animal (*M* = 4.78, *SD* = 2.30) were based upon personal relevance rating that we collected before ([39]) using the procedure described in the Ratings section below. As these ratings had only delivered one high salient category that we considered useful for online testing, we added a category (country of origin) for which we lacked relevance ratings, but that we reasoned to be of high personal relevance.

We followed the procedure of Dindo and Fowles [40] and asked participants to indicate how important, significant or relevant 12 different autobiographical categories are to them, including the details used in this experiment (see Table 1). They responded by choosing one option on a 9-point Likert scale (1 = not relevant at all, 5 = slightly relevant, 9 = absolutely relevant) using a drop-down menu.

### 3.3. Results and Discussion

#### 3.3.1. Results

As Table 1 shows, country of origin and birthday (*M* = 7.03, *SD* = 2.17) were rated as more salient than favourite colour and favourite animal (*M* = 5.55, *SD* = 2.09), *t*(250) = 11.71, *p* < .001, *d*_*within*_ = .74. We conclude that the saliency manipulation was successful.

All analyses were conducted with *R Studio* Version 0.98.945. The alpha level we used in all our analyses was .05. In our main analysis, we used a 2 (Identity knowledge: knowledgeable vs. naïve, between-subjects) by 2 (Stimulus: probe vs. irrelevant, within-subjects) by 2 (Saliency: salient vs. peripheral, within-subjects) mixed ANOVA on error rates and reaction times in milliseconds. We report effect size for the ANOVA using Cohen’s *f*, *f* = √[ηp2 /(1—ηp2)], and we used Cohen’s *d* for follow up contrasts [41]. We annotate Cohen’s *d* for within-subject and between-subject comparisons as *d*_*within*_ and *d*_*between*_. Following the recommendations of Lakens [42] and the meta-analysis of Suchotzki et al. [19], we calculated the probe-irrelevant within-subject contrast as *d*_*within*_ = *M(RT*_*(probes)*_ – *RT*_*(irrelevants)*_ / √(*SD*_*(probes)*_^*2*^ + SD_(irrelevants)_^2^ – 2*r*SD_(probes)_*SD_*(irrelevants)*_, where *r* is the Pearson correlation between *RT*_*(probes)*_ and *RT*_*(irrelevants)*_. Following the recommendations of Lakens [42] we calculated the between-subject contrast for knowledgeable versus naïve individuals as *d*_*between*_ = (*M*_*RT(Probe-Irrelevant Difference knowledgeable)*_—*M*_*RT(Probe-Irrelevant Difference naive)*_) / √(((*n*_*knowledgeable*_ – 1)*SD_*(Probe-Irrelevant Difference knowledgeable)*_^*2*^ + (*n*_*naive*_ – 1)*SD_*(Probe-Irrelevant Difference naïve)*_^*2*^)/*n*_*knowledgeable*_ + *n*_*naive*_ -2).

In addition to the group analysis, it is also interesting to examine individual classification accuracy. For individual diagnoses, we looked at the probe-irrelevant difference within each individual. Following Noordraven and Verschuere [17], we calculated the individual CIT score as follows: *d*_*CIT*_ = (*M*_*RT(probes)*_—*M*_*RT(irrelevants)*_) / *SD*_*RT(irrelevants)*_, and examined how well it performed as diagnostic criterion for individual knowledgeable/naive classification. Specifically, we used Receiver Operating Characteristics (ROC) curves. In ROC analysis, the specificity is set into relation to the sensitivity of the diagnostic measure. The overall performance of the criterion is the area under the curve (AUC) that can theoretically range from 0 to 1, whereby an AUC value of .5 indicates random classification [43]. We examined how well the individual Cohen’s d for the probe-irrelevant difference could discriminate knowledgeable from naive participants. All ROC calculations were conducted with the pROC package for R [44].

Trials where no response was recorded (i.e., RTs larger than 1500ms) were excluded from all subsequent analyses. For error rates, the 2 (Identity knowledge: knowledgeable vs. naïve) by 2 (Stimulus: probe vs. irrelevant) by 2 (Saliency: salient vs. peripheral) mixed ANOVA on error rates revealed a significant main effect of Stimulus, *F*(1, 201) = 7.31, *p* = .007, *f* = 0.19, and a significant interaction between Saliency and Stimulus, *F*(1, 201) = 4.68, *p* = .032, *f* = 0.15, that was of no further interest (it indicated that the probe-irrelevant difference was lower for low salient than for high salient items, irrespective of Identity knowledge). There was an interaction between Identity knowledge and Saliency, *F*(1, 201) = 4.57, *p* = .034, *f* = 0.15, and a three-way interaction between Identity knowledge X Saliency X Stimulus, *F*(1, 201) = 4.10, *p* = .044, *f* = 0.14. In order to grasp this three-way interaction, we conducted two separate 2 (Stimulus: probe vs. irrelevant) by 2 (Identity knowledge: knowledgeable vs. naive) mixed ANOVAs for high and low-salient items. For high-salient items, there was a significant main effect of Identity knowledge, *F*(1, 201) = 4.47, *p* = .036, *f* = 0.15, and a significant interaction between Identity knowledge and Stimulus, *F*(1, 201) = 8.79, *p* = .003, *f* = 0.21. The probe-irrelevant difference for knowledgeable participants (*M* = 0.68%, *SD* = 3.54%) was larger than for naïve participants (*M* = -0.49%, *SD* = 2.07%), *t*(201) = 2.96, *p* = .003, *d*_*between*_ = 0.39. For low-salient items, there was only a significant main effect of Stimulus, *F*(1, 201) = 7.93, *p* = .005, *f* = 0.20, indicating that error rates on probes were larger than on irrelevants (see Table 2).

**Table 2:** Mean reaction times (in ms) and mean error rates (in %; SDs in Parentheses) for low and high salient items in naïve and knowledgeable participants in Study1.

	Naive				Knowledgeable			
**RTs**								
	Probe	Irrelevant	Probe-Irrelevant difference	*d*_*within*_	Probe	Irrelevant	Probe-Irrelevant difference	*d*_*within*_
High salient	488 (47)	497 (43)	-9.43 (27.29)	-0.35	538 (51)	497 (43)	40.51 (34.31)	1.18
Low salient	503 (53)	483 (44)	19.55 (29.88)	0.65	500 (54)	483 (45)	16.63 (33.55)	0.50
Collapsed	495 (45)	490 (43)	4.64 (19.84)	0.23	519 (49)	490(43)	28.34 (27.49)	1.03
**Error rates**								
	Probe	Irrelevant	Probe-Irrelevant difference	*d*_*within*_	Probe	Irrelevant	Probe-Irrelevant difference	*d*_*within*_
High salient	0.63 (1.71)	1.12 (1.54)	-0.49 (2.07)	-0.24	1.67 (3.12)	0.98 (1.65)	0.68 (3.54)	0.19
Low salient	2.07 (5.99)	0.90 (2.29)	1.17 (5.15)	0.23	1.31 (4.12)	0.57 (1.01)	0.74 (4.26)	0.17
Collapsed	1.35 (3.14)	1.01 (1.60)	0.34 (2.74)	0.12	1.49 (2.61)	0.78 (1.08)	0.71 (2.76)	0.26

All trials with incorrect responses were excluded from the reaction time analysis as well as all trials with an RT below 150ms and above 800ms (cf [18]). The 2 (Identity knowledge: knowledgeable vs. naïve, between-subjects) by 2 (Stimulus: probe vs. irrelevant, within-subjects) by 2 (Saliency: salient vs. peripheral, within-subjects) mixed ANOVA on RTs in milliseconds revealed that significant main effects of Saliency, *F*(1, 201) = 58.09, *p* < .001, *f* = 0.54, of Stimulus, *F*(1, 201) = 103.28, *p* < .001, *f* = 0.72, and a significant interaction between Identity knowledge and Saliency, *F*(1, 201) = 61.19, *p* < .001, *f* = 0.55, and between Identity knowledge and Stimulus, *F*(1, 201) = 50.45, *p* < .001, *f* = 0.50. These effects were collapsed under the three-way significant interaction of Identity knowledge X Stimulus X Saliency, *F*(1, 201) = 83.58, *p* < .001, *f* = 0.64. Table 2 shows the RTs for each cell of our design. To narrow down the three-way interaction, we looked at the 2 (Stimulus: probe vs. irrelevant) by 2 (Identity knowledge: knowledgeable vs. naive) mixed ANOVA separately for high and low-salient items.

For high-salient items, the significant main effects of Stimulus, *F*(1, 201) = 51.68, *p* < .001, *f* = 0.51, and of Identity knowledge *F*(1, 201) = 16.60, *p* < .001, *f* = 0.29, subsumed under the significant Identity knowledge X Stimulus interaction, *F*(1, 201) = 133.42, *p* < .001, *f* = 0.81. This interaction indicated that the probe-irrelevant difference was greater for knowledgeable (*M* = 40.51 ms, *SD* = 34.31ms) than for naive participants (*M* = -9.43 ms, *SD* = 27.41 ms), *t*(201) = 11.55, *p* < .001, *d*_*between*_ = 1.64.

For low-salient items, there was only a significant main effect of Stimulus, *F*(1, 201) = 64.71, *p* < .001, *f* = 0.57, that is, RTs were larger for probes (*M* = 500.64 ms, *SD* = 52.12) than for irrelevants (*M* = 482.46 ms, *SD* = 43.62), *t*(202) = 8.28, *p* < .001, *d*_*within*_ = 0.58. The main effect of Identity knowledge and the crucial Identity knowledge X Stimulus effects were not significant, *F*s < 1.

The area under the curve for RTs was larger than for error rates, using DeLong's test for two ROC curves, *Z* = 4.25, *p* < .001, see Table 3. The 95% confidence interval of error rate AUC value includes .50 and is thus not significantly better than chance. For error rates, detection efficiency was higher for high salient items than for low salient item, *Z* = 2.82, *p* = .005. For RTs also, detection efficiency was higher for high salient items was significantly higher than for low salient items, *D* = 7.06, 2000 bootstraps, *p* < .001 using the bootstrap test for correlated ROC curves. The 95% confidence interval of the RT AUC value for low salient items includes .50 and is thus not significantly better than chance. In sum, significant detection at the individual level was restricted to the detection of high salient items, and RTs outperformed Error rates.

**Table 3:** Diagnostic efficiency of RTs and error rates in Study1.

	RTs		Error rates	
	ROC (95%CI)	Cohen’s *d*_between_	ROC (95%CI)	Cohen’s *d*_between_
High Salient	.88 (.83–.93)	1.59	.61 (.53–.68)	0.39
Low Salient	.52 (.44–.60)	-0.09	.45 (.38–.53)	0.09
Collapsed	.78 (.71–.84)	0.97	.57 (.50–.65)	0.13

#### 3.3.2. Discussion

The main goal of Study 1 was to examine the feasibility of online memory detection testing. We tested participants from diverse ethnic and geographic backgrounds. That we were able to test 255 participants for a modest reward in less than 12 hours, speaks to the efficiency of online testing. Importantly, replicating offline research, we found that reaction times could detect concealed memories at an accuracy that is well above chance [19]. Moreover, we also found that memory detection success was better for high salient items (country of origin and birthday) than for low salient items (favourite colour and favourite animal) replicating Verschuere, Kleinberg and Theocharidou [39]. In fact, memory detection was unsuccessful for low salient items. This finding, however, requires qualification, as it seems to be driven by significant probe-irrelevant difference for low salient items in naïve participants (+19.55 ms, *d*_within_ = +0.65; which is indicative of a bias in the test) rather than the lack of such a probe-irrelevant difference for low salient items in knowledgeable participants. To comprehend these results, we ran supplementary analyses on the category level. These analyses showed that the bias arose from the category *favourite animals* with naive participants reacting slower to the probe animal than to the irrelevant animals (perhaps because their probe animal HORSE resembled their target animal MOUSE more so than the irrelevant animals ELEPHANT, FERRET, WHALE, RABBIT). After exclusion of the animal category, the mean Cohen’s *d*_*CIT*_ for high-salient items (*M*_*naive*_ = -0.09, *SD*_*naive*_ = 0.27; *M*_*knowledgeable*_ = 0.43, *SD*_*knowledgeable*_ = 0.38) was still larger than for low-salient items (*M*_*naive*_ = 0.02, *SD*_*naive*_ = 0.37; *M*_*knowledgeable*_ = 0.14, *SD*_*knowledgeable*_ = 0.51). While our main analyses confirmed our key prediction, we decided to run an additional study to rule out that moderation of memory detection success by item saliency would be due to a biased test.

## Experiment 2

The overall aim of Study 2 is identical to that of Study 1. We examined the feasibility of online memory detection testing, and investigated whether item saliency moderates memory detection success. The Method of Study 2 is identical to that of Study1, with the following exceptions. First, and most importantly, we randomly assigned items to be probe, target, or irrelevant items in naive participants, and randomly assigned items to be target or irrelevant items in knowledgeable individuals. Second, we abbreviated country names. In Study1, we used full country names. As a result, North-American individuals received a probe item—*United States of America* – that was substantially longer than the irrelevant items *Japan*, *Peru*, *Poland*, or *Sweden*, which could have artificially inflated the Saliency effect (i.e., greater memory detection success for high salient than for low salient items). Although additional analyses in non-US participants only replicated the moderation by saliency effect, we decided to abbreviate the most common probe-country to U.S.A. in Study 2. Third, we inserted an additional validity check: If the RT on more than 20% of the trials in any of the practice phases was lower than 150ms, we concluded that the user must have pressed a button continuously. The participants saw a warning message and had to do the relevant practice phase again. The study was approved by the ethical committee of the Department of Psychology of the University of Amsterdam (2013-CP-3053).

### 4.1. Materials and Method

**4.1.1. Participants**. Similar to study 1, the 250 HITs resulted in 262 completed tests, of which we were missing data from 3 participants. Our sample consisted of 259 participants (53% females; *M*_*age*_ = 34.94 years, *SD*_*age*_ = 11.85). Compensation and completion time were equal to study 1. Education was distributed as follows: 46% college education, 30% university, 9% professional training, 15% high school, and 0.3% elementary school. English was the most common language (70%), followed by Indian languages (19%) and Mandarin, German, Chinese, Others, and Spanish (together 11%). Participants originated mostly from the US (57.8%) and India (33.6%), but also from 9 other countries (American Samoa, Aruba, Bangladesh, Canada, Georgia, Peru, Philippines, Sweden, Tanzania, together 8.6%). There were five duplicate IP addresses and two additional IP addresses that were found in Study 1 (leaving 247 participants). We excluded all data of these IP addresses from analysis. Thirty-five participants were excluded based on having 50% errors or more on any of the three trial types (probe, target, irrelevant). Subject loss was 19.84%, resulting in a final sample of 212 participants. 111 participants were randomly assigned to the naive identity knowledge condition (52% females, *M*_*age*_ = 35.96 years, *SD*_*age*_ = 12.04) and 100 to the knowledgeable condition (49% females, *M*_*age*_ = 35.16 years, *SD*_*age*_ = 12.25). There was no difference in gender *X* ^*2*^(1) < 1, *p* = .789, or age *t*(210) = 0.48, *p* = .631.

### 4.2. Results and Discussion

#### 4.2.1. Results

Table 4 shows that the significance ratings for country of origin and birthday (*M* = 7.36, *SD* = 1.92) were higher than those of the categories favourite colour and favourite animal (*M* = 5.70, *SD* = 1.98), *t*(260) = 11.805, *p* < .001, *d*_*within*_ = 0.73. We conclude that the Saliency manipulation was successful.

**Table 4:** Relevance Ratings of Different Categories of Autobiographical Information in Study2 (presented in descending order).

Category	*Mean*	*SD*
Country of Origin	7.44	2.07
Birthday (Day and Month)	7.28	2.18
Age in years	6.59	2.31
Favorite animal	5.85	2.31
Favorite color	5.55	2.32
Political preference	4.77	2.52
Favorite sports	4.61	2.68
Favorite holiday destination	4.42	2.61
Favorite sex position	4.15	2.84
Favorite car	4.13	2.53
Favorite sex location	3.77	2.69
Favorite celebrity	3.54	2.50
Favorite international author	3.46	2.33
Favorite alcoholic drink	3.33	2.25
Favorite drug	2.49	2.26

Trials where no response was recorded (i.e., RTs larger than 1500ms) were excluded from all subsequent analyses. The 2 (Identity knowledge: knowledgeable vs. naïve) by 2 (Stimulus: probe vs. irrelevant) by 2 (Saliency: salient vs. peripheral) mixed ANOVA on error rates indicated a significant main effect of Stimulus, *F*(1, 210) = 10.14, *p* = .002, *f* = 0.22, and a significant interaction between Identity knowledge and Stimulus, *F*(1, 210) = 6.33, *p* = .013, *f* = 0.17. All other effect were non-significant with *F* < 1. The 2-way interaction indicated that the probe-irrelevant difference was higher for knowledgeable participants (*M* = 1.11%, *SD* = 3.44%) than for naive participants (*M* = 0.13%, *SD* = 2.15%), *t*(210) = 2.51, *p* = .013, *d*_*between*_ = 0.35 (see Table 5).

**Table 5:** Mean reaction times (in ms) and mean error rates (in %; SDs in parentheses) for low and high salient items in naïve and knowledgeable participants in Study 2.

	Naive				Knowledgeable			
	**RTs**							
	Probe	Irrelevant	Probe-Irrelevant difference	*d*_*within*_	Probe	Irrelevant	Probe-Irrelevant difference	d_within_
High salient	502 (45)	500 (42)	2.03 (28.69)	0.07	537 (50)	497 (46)	39.28 37.25)	1.05
Low salient	491 (49)	495 (43)	−3.66 (31.13)	−0.12	508 (55)	492 (47)	15.41 (35.01)	0.44
Collapsed	497 (43)	497 (41)	-0.80 (21.63)	−0.04	522 (48)	495 (46)	27.41 (28.61)	0.96
	**Error rates**							
	Probe	Irrelevant	Probe-Irrelevant difference	*d*_*within*_	Probe	Irrelevant	Probe-Irrelevant difference	*d*_within_
High salient	1.04 (2.95)	0.95 (1.66)	0.07 (3.27)	0.02	1.64 (3.64)	0.55 (1.01)	1.10 (3.80)	0.29
Low salient	0.86 (3.22)	0.66 (0.99)	0.19 (3.36)	0.06	1.70 (5.73)	0.57 (0.93)	1.13 (5.83)	0.19
Collapsed	0.95 (2.08)	0.81 (1.01)	0.13 (2.15)	0.06	1.67 (3.37)	0.56 (0.70)	1.11 (3.44)	0.32

The 2 (Identity knowledge: knowledgeable vs. naïve, between-subjects) by 2 (Stimulus: probe vs. irrelevant, within-subjects) by 2 (Saliency: salient vs. peripheral, within-subjects) mixed ANOVA on correct RTs between 150–800ms indicated that with exemption of the main effect of Identity knowledge, all main effects and interactions were significant. These effects subsumed under the significant three-way interaction between Identity knowledge, Saliency and Stimulus, *F*(1, 210) = 9.54, *p* = .002 *f* = 0.21. We looked at the interaction between Stimulus and Identity knowledge separately per item Saliency.

For high-salient items, there were significant main effects of Identity knowledge, *F*(1, 210) = 7.49, *p* = .006, *f* = 0.19, and of Stimulus, *F*(1, 210) = 82.66, *p* < .001, *f* = 0.63. The significant interaction between Identity knowledge and Stimulus, *F*(1, 210) = 67.18, *p* < .001, *f* = 0.57, revealed that the probe-irrelevant difference was larger for knowledgeable participants (*M* = 39.28, *SD* = 37.25) than for naive participants (*M* = 2.03, *SD* = 28.69), *t*(210) = 8.20, *p* < .001, *d*_*between*_ = 1.13.

For low-salient items, there was a significant main effect of Stimulus, *F*(1, 210) = 6.69, *p* = .010, *f* = 0.18, and a significant interaction between Stimulus and Identity knowledge, *F*(1, 210) = 17.62, *p* < .001 *f* = 0.29. The interaction indicated that the probe-irrelevant difference was larger in knowledgeable (*M* = 15.41, *SD* = 35.01) than in naive participants (*M* = −3.66, *SD* = 31.13), *t*(210) = 4.20, *p* < .001, *d*_*between*_ = 0.58 (see Table 5).

The overall AUC for RTs was higher than that for error rates, *D* = 7.42, 2000 bootstraps, *p* < .001, see Table 6. For RTs, the AUC for high salient items was significantly larger than for low salient items, *Z* = 2.55, *p* = .011. For error rates there was no difference between high and low salient items, p > .05.

**Table 6:** Diagnostic efficiency of RTs and error rates in Study2

	RTs		Error rates	
	ROC (95%CI)	Cohen’s *d*_between_	ROC (95%CI)	Cohen’s *d*_between_
High Salient	.79 (.72–.85)	1.13	.45 (.37–.52)	0.29
Low Salient	.67 (.60–.74)	0.58	.43 (.36–.50)	0.20
Collapsed	.80 (.74–.86)	1.12	.41 (.36–.50)	0.35

#### 4.2.2. Discussion

Using an optimised procedure that provided better safeguards against item biases, the results of Study 2 replicated those of Study 1, and thereby confirm that memory detection using reaction times can be validly and efficiently conducted online. High salient items being more easily detected than low salient items.

#### 4.2.3 Supplementary analysis: On the inclusion of RTs that exceed the response deadline

Although not the focus of the present article, we investigated whether including RTs beyond the 800ms response deadline would increase diagnostic efficiency. For these exploratory analyses we used the combined samples of Study1 and Study2. Whereas our lab has typically discarded all RTs beyond the response deadline [17][18], the Seymour lab [9][10][11][12] also makes us of a response deadline but includes RTs beyond the response deadline to a max RT of 1500ms. We reasoned that this is precisely an issue that can be investigated with well-powered studies such as the present one. We reran the key analyses with inclusion of RTs between 800 and 1500ms. We were interested whether the diagnostic power of the memory detection test would increase, so we focused upon the ROC analyses. The overall *AUC* was .77 (*95% CI*: .72–.81; high salient items: *AUC* = .80, *95% CI*: .76–.85; low salient items: *AUC* = .59, *95% CI*: .53–.64), and did not differ from the one that excludes RTs above the response deadline, *Z* = 1.24, *p* = .216. We conclude that including larger RTs does not add to the diagnostic power of the CIT.

#### 4.2.4. Supplementary analysis: Reliability of the online CIT

We also examined the reliability of our online CIT in order to grasp how much noise the online procedure adds to the test. We calculated the Spearman-Brown split-half reliability of the individual Cohen’s *d*_*CIT*_ values for both experiments with the following formula: *ρ* = 2*r* / (1+*r*), whereby *r* is the Pearson correlation between the odd-numbered trials and the even-numbered trials [45][46]. For Experiment 1 the split-half reliability was *ρ* = .39 (*95% CI*: .26–.50) for naïve participants and *ρ* = .51 (*95% CI*: .40–.60) for knowledgeable participants. The corresponding values of Experiment 2 were *ρ* = .35 (*95% CI*: .23–.47) and *ρ* = .67 (*95% CI*: .59–.74) for naïve and knowledgeable participants, respectively.

## General Discussion

Our aim was to investigate the potential of online memory detection to conduct well-powered memory detection studies in an efficient and valid way. Using the first online RT-based memory detection test, we assessed participants on low and high salient autobiographical items. The efficiency of this first web-based memory detection is apparent from the fact that we could test a large group of people—from diverse ethnic and geographic backgrounds—at a low cost in a short period of time. Clearly, efficiency alone is not enough.

### 5.1. Memory detection 2.0: Validity

The results of our web-based memory detection test seem generally in line with previous findings obtained in offline laboratory studies. First, as may be expected by being a RT-based test, the diagnostic efficiency of RTs outperformed that of error rate. Error rate was generally very low for probe and irrelevant items, and floor effect may render error rates to result in low validity. We therefore focus our discussion upon RTs. Second, the effect size of RT-based memory detection in knowledgeable individuals was large (Study1: *d*_*within*_ = 1.03; Study2: *d*_*within*_ = 0.96). The effect size obtained in both studies for knowledgeable individuals fell within that observed in offline RT-CIT research (*d*_*within*_ = 1.12 [95% CI: 0.84, 1.39]). Our web-based findings seem to corroborate the idea that the RT-based memory detection effect in knowledgeable individuals is large. This is an important observation as earlier laboratory studies relied upon small samples, and there were indications of publication bias [19]. Third, the validity of memory detection is moderated by item saliency, as has been found before in laboratory studies [34][35][36][37][38][47]. Detection efficiency for high salient items was higher than for low salient items in both studies. In Study1, RT-based memory detection for low salient items was at chance level, which might have been related to item bias. Study2 prevented such bias and found that detection efficiency was also significant for low salient items. Thus, while its clear that detection efficiency for high salient items is better than for low salient items, it seems important to study under which conditions low salient items can also be detected.

### 5.2. Limitations

This study is not without its limitations, most related to online testing. First, online testing may bring about more noise in the data. Participants use their own computer and Internet connection, which are likely to differ considerably in processing efficiency and may have affected the presentation and measurement precision. Also, we do not know to what extent participants were focused on our test or whether they were also engaged in other activities (eating, listening to music, surfing the web, etc). Note, however, that we do not compare mean RTs between participants, but that the key comparison – for both knowledgeable and naïve participants – involves a within-subject comparison (probe RT versus irrelevant RTs). Moreover, we used three successive practice phases that assured understanding and adherence to the test instructions, and we used strict validity checks as exclusion criteria. Under these conditions, the validity of the online RT-CIT appeared to be similar to that obtained in laboratory RT-CIT studies. Much less is known about the reliability of the RT-CIT. We know of one paper that reports upon the reliability of the RT-CIT [17], which reports a split-half correlation of *r* = .38, which equals *ρ* = .55 (*95% CI*: .30–.73). With *ρ* = .51–.67 in knowledgeable individuals, the reliability in our online RT-CIT seems also comparable to that observed in the lab.

Second, anticipating sensitivity with regard to privacy, we used categories that have not often been used in offline memory detection research (birthday being the exception). Our subjective ratings and memory detection results show that this did not prevent us from creating high versus low salient categories. Still, the subjective ratings indicate that it is difficult to come up with categories that are as significant as those typically used in offline memory detection (e.g., participant’s name, names of relatives, loved ones or friends, address, social security number, phone number, address). The recommendation to use at least five high salient items [48] may be difficult to achieve in online autobiographical memory detection testing.

Third, the validity of this study relies upon honest completion of the autobiographical details at the beginning of the study (country of origin, birthday, favourite colour, favourite animal). In offline research, the experimenter may try and verify the provided information, although there too it is not always possible and not always done (for an exception see [49]). Importantly, researchers have found through consistency checks that respondents on Amazon Mechanical Turk are generally honest about the provided demographical information [50][51][52].

### 5.3. Future research

Given that this study is the first to take memory detection to the web, there is still much to be learned. First, while we successfully detect autobiographical information, it is important to examine whether one can also detect a hidden card, memorised code items, and particularly mock crime details [5].

Second, while detection efficiency was significantly above chance, individual classification accuracy was lower than what has sometimes been observed in the laboratory (AUCs > .90 see [10]; [14]; [16]). Such between-studies comparison is hazardous, and a direct comparison between online and offline memory detection is needed. While our findings corroborate the idea that the results of online research generalize to those observed in the laboratory [30][31][32], it will be important for future research to directly address the comparability between online and offline RT-CIT research. Such a direct comparison has been conducted for other RT-based tests. Houben and Wiers [53] examined the effects of testing environment (lab vs. home) and software (Inquisit [regular lab software] versus Flash [regular software for online testing]) on the validity of implicit alcohol associations measured in the RT-based Implicit Association Test (IAT). There were no effects of either testing environment or software, paving the way for online RT research. Still, it is important to examine whether these findings also hold for the RT-CIT and to run a direct comparison of the web-based CIT and the lab-based CIT.

Third, it is interesting to see whether factors that have been shown to moderate offline memory detection (e.g., number of items, motivation to conceal, faking [5][6][2], also moderate online memory detection success. The replication of the moderating role of item saliency in the present study indicates that this is indeed a fruitful avenue for future research.

Fourth, several methodological issues have not been empirically investigated yet, and online testing seems to provide an interesting platform to study those issues. As a first example, we investigated whether or not one should include RTs beyond the response deadline, and found that it does not add to the diagnostic power of the test. Memory detection 2.0 can rapidly shed light on such and related issues: How many trials are needed for a reliable and valid test result? What is the optimal presentation duration, ISI, and response deadline? Do pictures and words work equally well? What is test-retest reliability? One of the reasons why researchers have not thoroughly investigated these questions is that they carefully balance their use of resources. Because online research is much more efficient, one can now start to answer these basic, yet important questions.

Fifth, memory detection 2.0 opens new possibilities such as remote testing (i.e., the examiner not physically being present with the examinee). Remote testing in the forensic context will need a means to guarantee the identity of the examinee taking the test. Still, remote testing may be of use, because for instance the police can assure that it is the actual suspect taking the test, and the examiner does not need to physically be present. Remote testing may also be beneficial for simultaneous testing. Note that simultaneous testing is also possible with physiological measures (see e.g., [54], but becomes much easier with online memory detection.

## Conclusions

In two studies, encompassing more than 500 participants, we show that memory detection with RTs can be efficiently and validly run online. As in offline laboratory studies, we found that RTs outperform error rates, that RT-based memory detection results in large effects, and that item saliency moderates this effect. Online testing seems an exciting new method to efficiently address old and new questions related to memory detection.
